# Automated and Scalable Verification of Integer Multipliers

**DOI:** 10.1007/978-3-030-53288-8_23

**Published:** 2020-06-13

**Authors:** Mertcan Temel, Anna Slobodova, Warren A. Hunt

**Affiliations:** 8 Microsoft Research Lab, Redmond, WA USA; 9 University of Southern California, Los Angeles, CA USA; 10 University of Texas at Austin, Austin, TX USA; 11 Centaur Technology, Inc., Austin, TX USA

**Keywords:** Multipliers, Hardware verification, Formal methods, ACL2

## Abstract

The automatic formal verification of multiplier designs has been pursued since the introduction of BDDs. We present a new rewriter-based method for efficient and automatic verification of signed and unsigned integer multiplier designs. We have proved the soundness of this method using the ACL2 theorem prover, and we can verify integer multiplier designs with various architectures automatically, including Wallace, Dadda, and 4-to-2 compressor trees, designed with Booth encoding and various types of final stage adders. Our experiments have shown that our approach scales well in terms of time and memory. With our method, we can confirm the correctness of $1024\times 1024$-bit multiplier designs within minutes.

## Introduction

Arithmetic circuit designs may contain bugs that may not be detected through random testing. Since the Pentium FDIV bug [29], formal verification has become more prominent for validating the correctness of arithmetic circuits. Despite being a crucial part of all processors, verifying the correctness of arithmetic circuits, specifically multipliers, is still an ongoing challenge.

There have been numerous efforts to find a scalable and automated method to formally verify integer multipliers. Early methods that were based on attempts to represent hardware and its specification in various canonical forms - BDDs [6] and derivatives, have an exponential space complexity. Therefore, they were applicable only for small circuits. Similarly, SAT-based methods did not prove to be scalable [28].

There are several approaches for the verification of hardware multipliers used in the industry. One is based on writing a simple RTL multiplier design without optimizations and comparing it to the candidate multiplier design through equivalence checking [14, 35]. This approach works only when the reference design is structurally close to the original under verification and relies on the correctness of the reference design and proof maintenance whenever designers make structural changes. Another approach is to find a suitable decomposition of a design into parts that can be verified automatically and compose those results into a top-level theorem [13, 15, 30]. The drawback of this method is that it requires manual intervention by the verification engineer who decides about the boundaries of the decomposition. A third approach involves guiding a mechanized proof checker manually [27].

In recent years, the search for more automatic procedures resulted in methods based on symbolic computational algebra [7, 16, 22, 23, 40] . This approach makes it possible for certain types of multipliers to be verified automatically for larger designs. However, they have limitations as to what type of multipliers they can check (see experiments in Sect. 6). They are implemented as unverified programs and, as far as we are aware, only one of them [16] produces certificates.

We have developed an automatic rewriter-based method for verification of hardware integer multipliers that is

- widely applicable,
- provably correct, and
- scalable

We implemented and verified our method with the ACL2 theorem proving system, which is a subset of the LISP programming language. Our method is not ACL2 specific and can be adapted to other platforms with suitable adjustments. In this paper, we also provide proof of its termination. Even though we have not proved the completeness of this method, our tool can verify various multiplier designs. We test our method on designs implemented with (System) Verilog where design hierarchy is maintained. We can verify various types of multipliers in a favorable time; for example, we tested our method with 8 different types of $1024\times 1024$ multipliers and verified each of them in less than 10 min, while the other state-of-the-art tools ran for more than 3 h.

The paper is structured as follows. In Sect. 2, we present some concepts that might be necessary to understand our approach. These include the basic notion of term rewriting and the ACL2 system (Sect. 2.1), the semantics for hardware modeling (Sect. 2.2), and some basic multiplier architectures (Sect. 2.3). Preliminaries are followed by our specification and top-level correctness theorem for multiplier designs (Sect. 3). We explain our methodology to prove this top-level correctness theorem with term rewriting in Sect. 4. Section 5 describes the termination of our rewriting algorithm. Experiments with various benchmarks are given and discussed in Sect. 6.

## Preliminaries

In this section, we describe the concepts and tools required to understand the method proposed in this paper. We review the ACL2 theorem prover and term rewriting, how Verilog designs are translated and used in proofs, and various integer multiplier architectures.

### ACL2 and Term Rewriting

ACL2 is a LISP-based interactive theorem prover that can be used to model computer systems and prove properties about such models using both its internal procedures as well as appealing to external tools such as SAT and SMT solvers. ACL2 is used by the industry for both software and hardware verification [12]. Our methodology to prove multipliers correct uses ACL2-based term rewriting.

ACL2 can store proved lemmas as *rewrite rules*, and later use them when attempting to confirm other conjectures. ACL2 terms are prefix expressions and rewriting is attempted on terms such as  . Left-hand side of a rewrite rule is unified with terms; in case of a successful unification, the matched term is replaced by a properly instantiated right-hand side if all hypotheses are satisfied. Example 1 shows two rewrite rules, the second of which can be proved using the first as a lemma. When users submit a  event, ACL2 attempts to confirm the conjecture by rewriting it in an inside-out manner. For the conjecture given in  , the rewriter replaces (+ x (- x)) and (+ y (- y)) with 0 using a-a as a lemma. Then the resulting term (+ 0 0) is replaced with 0 using the executable counterpart of the function +.

#### Example 1

A simple rewrite rule  , and a theorem  proved subsequently using  as a lemma. 
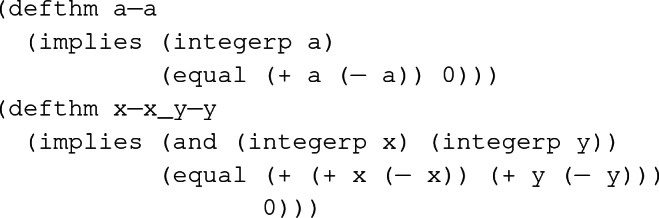


The rewriting mechanism in ACL2 is much more complex and intricate than we indicate here [18]. Throughout the rest of this paper, we omit ACL2 specific implementation details whenever possible. Understanding the basics of term rewriting is sufficient to follow our methodology.

### Semantics for Hardware Designs

We convert (System) Verilog designs to *SVL* netlists in ACL2 and use SVL functions for semantics and simulation of circuit designs [33]. SVL netlists preserve hierarchical information about hardware designs and they are based on the *SV* [31] and *VL* [32] tools that are also included in the ACL2 libraries. These tools have been used by several companies to confirm the correctness of various circuit designs [12]. In this section, we describe the format of SVL netlists, and how they are simulated hierarchically.

An SVL netlist is an association list where each key is a module name, and its corresponding value is the definition of the module. An SVL module is composed of input and output signals, and a list of occurrences. An occurrence can be an assignment or an instantiation of another module. Example 2 shows a simplified SVL netlist containing a half and a full-adder.

#### Example 2

An *SVL* netlist for half and full-adder. 
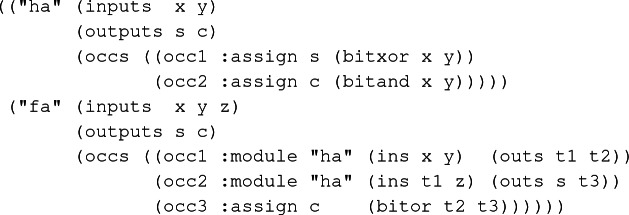


The semantics of an SVL netlist is given by a recursively defined ACL2 function,  . This function traverses occurrences of a module and simulates them in order by evaluating the assignments and making a recursive call for the submodules. After each occurrence, the values of wires/signals are stored in an association list, and when finished,  retrieves and returns the values of output signals from this association list. These values can be concrete (svl-run is executed), or symbolic (the rewriter processes a call of svl-run with variables for inputs), which can create ACL2 expressions representing the functionality of the design for each output. For example, we can generate expressions for the outputs of the full-adder ("fa") in Example 2:  and  . Alternatively, since the design retains hierarchy, submodules can be replaced by their specification. For example, assume that we have specification functions  and  for each output of the half-adder ("ha"), and we proved a rewrite rule to replace calls of svl-run of "ha" with these functions. If we rewrite the instantiations of "ha" with this rule while expanding the definition of "fa", we can instead get  and  for each output of "fa".

### Multiplier Architectures

In this section, we discuss the most commonly used algorithms to implement integer multipliers. We summarize partial-product *generation* algorithms, such as Booth encoding, and partial-product *summation* algorithms, such as Wallace-tree. Even though the applicability of our verification method is not confined to a specific set of algorithms, reviewing them is beneficial for understanding the verification problem.

**Fig. 1.**
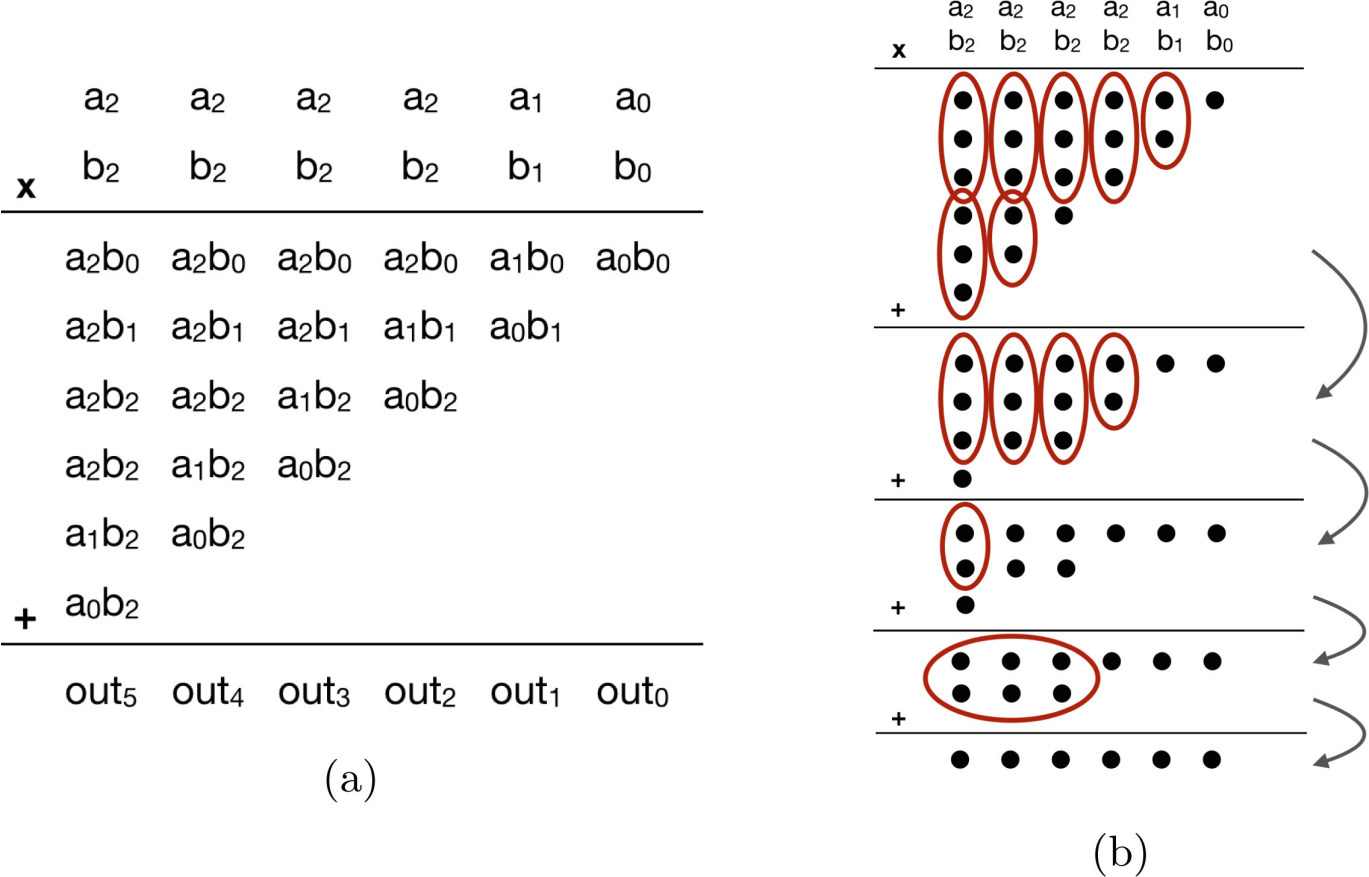
(a) Grade-school-like multiplication for two 3-bit two’s-complement integers, and (b) a Wallace-tree-like multiplier performing bit-level additions on the partial products

We can divide multiplier designs into two main components: partial product generation and summation. Figure 1a shows these two steps on multiplication of two 3-bit two’s-complement signed integers. We perform sign-extension (for signed numbers) or zero-extension (for unsigned numbers) on inputs, generate partial products, and then add them together to obtain the multiplication result in a fashion similar to grade-school multiplication. The integer multipliers we have verified implement various partial-product generation and summation algorithms for the same functionality with optimizations for better gate-delay and/or area.

Baugh-Wooley [1] and Booth [2] are commonly used algorithms to generate partial products. Baugh-Wooley is used for signed multiplication, and it generates partial products as shown in Fig. 1a, but with a sign-extension algorithm to prevent the repetition of generated partial product bits. A more commonly used alternative is Booth encoding, which can be used for both signed and unsigned multiplication. Instead of simply multiplying all the single bits of the two inputs with each other, Booth encoding uses more than one bit at a time from one of the operands, and it derives a more complex form for partial products. This helps reduce the number of rows for partial products, thus helping shrink the summation circuitry and allowing more parallelism. Booth encoding can be implemented with different radices, which determine the number of multiplier bits used at a time to create partial products (e.g., Booth radix-4 [21] uses 3 bits at a time). The higher the radix, the fewer the partial products; however, higher radices yield a more complex design. Booth encoding can be combined with sign-extension algorithms [38] to prevent repetition in generated partial products.

A rudimentary way to sum partial products is by using a shift-and-add algorithm. One may use an accumulator and a vector adder such as a ripple-carry adder to shift and add partial products. An array multiplier is a variation of this algorithm and it is implemented using a very similar principle with some additional optimizations. Due to their regular structure, verifying the correctness of these multipliers has not been a challenging problem [5]. However, these circuits often have very large gate delays, and Wallace-tree like multipliers are preferred over these algorithms in industrial applications.

A family of partial product summation algorithms, which are often called Wallace-tree like multipliers [36], use parallelism to obtain multiplication results with less gate-delay but produce a very irregular and complex design structure. Figure 1b shows an example of a Wallace-tree algorithm. In the first summation layer, we see the generated partial products corresponding to the ones in Fig. 1a. The Wallace-tree algorithm selects groups of bits from these partial products and passes them to full and half-adders. After these parallel bit-level additions, resulting carry and sum output bits are replaced on another layer whose summation will also yield the multiplication result. At each stage, layers are compressed, and the number of rows decreases. We repeat this process until we reach a state where we have only two rows. Then, instead of using full and half adders to finish additions, a vector adder (final stage adder), such as carry-lookahead and parallel prefix adders, is used. This method may provide a significant delay reduction over array multipliers. There exist numerous variations of Wallace-tree multipliers such as Dadda-tree [8] and 4-to-2 compressor trees [11]. Due to their highly irregular structure, reasoning about Wallace-tree like multipliers is a difficult problem, especially when combined with complex partial product generation algorithms such as Booth encoding. There is a lot of room for circuit designers to deviate from text-book algorithm definitions when creating multipliers, which increases the importance of having an automated method to verify these circuits with minimal assumptions about the structure.

## Specification

We aim to prove the functional correctness of signed and unsigned multiplier designs. We do that by proving an ACL2 theorem demonstrating the equivalence of semantics of a multiplier circuit design to the built-in ACL2 multiplication function (*) with appropriate sign extensions and truncations.

We work with integer multiplier circuits that are designed to multiply two numbers (signed or unsigned) stored in bit-vectors and cut (truncate) the resulting number to return it as a bit-vector. If we are multiplying *m*-bit and *n*-bit numbers, then the first $m+n$ bits of the result is sufficient to represent all output values. For example, assume that we are multiplying signed numbers –4 and 3, represented with 4-bit vector  and 3-bit vector  , respectively. Then, a correct multiplier would return the 7-bit vector  , which represents -12.

Listing 1.1 shows the final ACL2 theorem we prove for signed integer multipliers, where *a* and *b* are variables and $$*m*$$ and $$*n*$$ are concrete values^1^. This theorem states that for all integers *a* and *b*, simulating an *m*-by-*n* signed multiplier circuit returns a value that is equivalent to multiplication of sign-extended *a* and *b*, truncated at $$m + n$$ bits. On the left-hand side,  is an ACL2 constant that contains the multiplier design in SVL format which is translated from (System) Verilog, and  is the function to simulate this module with inputs *a* and *b*. On the right-hand side, * is the built-in integer multiplication function,  returns first $$m + n$$ bits of the result, and  returns a number that represents the sign-extended value of a bit-vector. Multiplier designs are implemented with fixed values of *m* and *n*; therefore, we prove such theorems for constants *m* and *n* and variables *a* and *b*. The ACL2 theorem for unsigned multiplication has the same form but in the place of  , we use the  function, which performs zero-extension. The actual statement of the theorem contains more components than shown, including function calls to extract outputs and parameters for state-holding elements; we only give the essentials for brevity.
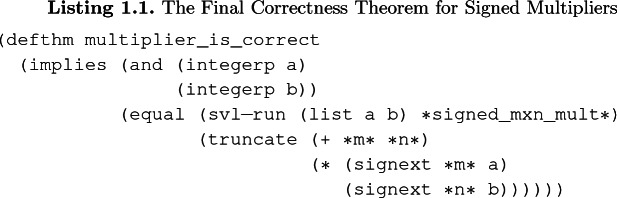


## Methodology

The correctness theorem given in Listing 1.1 is proved by rewriting both sides of the equality to two syntactically equivalent terms. In this section, we describe our methodology to rewrite both sides to a specific form through an automated rewriting mechanism.

We have a targeted final expression for each output bit of a multiplier design, the mathematical formula of which is given in Definition 2. The variables *a* and *b* are the inputs/operands of multiplication with a certain size (e.g., 64 bits for 64 $\times$ 64 multiplication); and in this formula, they are sign-extended for two’s complement signed multiplication or zero-extended for unsigned multiplication.

### Definition 1

We define functions *s* and *c* as follows.

$$\begin{aligned} \forall x \in \mathbb {Z} \ s(x)&= mod_2 (x) \\ \forall x \in \mathbb {Z} \ c(x)&= \Bigl \lfloor {\frac{x}{2}} \Bigr \rfloor \end{aligned}$$

### Definition 2

The targeted form for each output bit ($out_j$) is defined as follows.

$$ \begin{alignedat}{2} w_j =&{\left\{ \begin{array}{ll} {(\sum \limits _{i=0}^{j} {a_i b_{j-i}}) + c(w_{j-1})} &{} \quad \text {if j} \ge 0\\ 0 &{} \quad \text {otherwise.} \\ \end{array}\right. } \\ out_{j} =&\ s(w_{j}) \end{alignedat}$$

where $a_ib_{j-i}$ is logical AND of the *i*th and $(j-i)$th bits of operands *a* and *b*.

Table 1 shows an example of this targeted final form for the first four output bits of $3\times 3$ two’s complement signed multiplication (see Fig. 1a). Each output bit is represented with expressions composed of the *s*, *c*, and $+$ functions. In this representation, the outermost function of each expression is *s*, carry bits from previous columns are calculated with a single *c* per column, and the terms in summations are sorted lexicographically. Two’s complement signed or unsigned integer multiplication implemented by our candidate designs (See Sect. 6) can be represented by an expression of this form.

**Table 1.** Expressions for the final form of the first four output bits from Fig. 1a

$out_3$	$out_2$	$out_1$	$out_0$
$$\begin{array}{c} s(a_0 b_3 + a_1 b_2 + a_2 b_1 + a_3 b_0\\ +c(a_0b_2 + a_1b_1 + a_2b_0\\ +c(a_1b_0 + a_0b_1\\ +c(a_0b_0))) \end{array} $$	$$\begin{array}{r} s(a_0 b_2 + a_1 b_1 + a_2 b_0\\ +c(a_1b_0 + a_0b_1\\ +c(a_0b_0)))\\ \end{array} $$	$$\begin{array}{r} s(a_1b_0 + a_0b_1\\ +c(a_0b_0 ) )\\ \end{array} $$	$$\begin{array}{l} s(a_0b_0 )\\ \end{array}$$

**Fig. 2.**
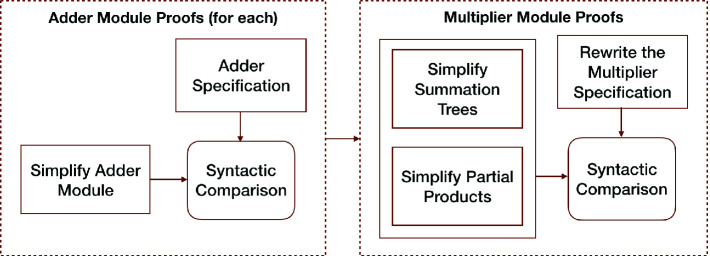
Summary of the overall method

A summary of our rewriting approach to verify multiplier designs is given in Fig. 2. Our method works with design semantics such as SVL where circuit hierarchy can be maintained and we reason about adder modules and the main multiplier module at different stages. As the first step, we work only with adder modules (e.g., half/full-adders and final stage adders) instantiated as submodules by the candidate multiplier design. We state a conjecture similar to Listing 1.1 for each adder module. We simplify their gate-level circuit description and prove them equivalent to their specification. We save these proofs as rewrite rules where *lhs* is svl-run of adder module and *rhs* is its specification. Having created these rewrite rules for all the adder modules, we start working on the correctness proof of the multiplier design as stated in Listing 1.1. On the LHS, as we derive ACL2 expressions from the definition of multiplier designs (see Sect. 2.2), we replace instantiated adder modules with their specification, and we apply two other sets of rewrite rules to simplify summation tree and partial product logic. On the RHS, we rewrite the multiplier specification into the targeted final form of multiplication, and we syntactically compare the two resulting terms to conclude our multiplier design proofs.

We simplify adder and multiplier modules by stating a set of lemmas in the form of equality $lhs = rhs$. These lemmas are used to create a term rewriting mechanism where expressions from circuit definitions are unified with *lhs* and replaced with their corresponding *rhs*. We aim to provide a set of lemmas so that such an automated system of rewriting can reduce a wide range of multiplier circuit designs to the final form as given in Table 1. In pursuit of this goal, we devised and experimented with various rewriting strategies; and we came up with a well-performing heuristic. In the subsections below, we describe these lemmas separated into two main sets for adder and multiplier modules, and the general mechanism to prove them equivalent to their specification. The lemmas we introduce are proved using ACL2, and we omit the proofs for brevity.

### Adder Module Proofs

The first step of our rewriting strategy is to represent the outputs of adder modules in terms of the *s*, *c*, and $+$ functions. We first determine the modules that serve as adder components in multiplier designs, such as half-adders, full-adders, 4-to-2-compressors, and final stage adders. Then we state a conjecture similar to Listing 1.1 where *lhs* is  of the adder module and *rhs* is its specification. We prove this conjecture with a library of rewrite rules, derived from the lemmas given in this section, which can simplify various types of adder modules and prove them equivalent to their specification.

For vector adders, specifications have a fixed format as shown in Table 2; however, for single-bit adders, such as full-adders and 4-to-2 compressors, specifications may vary. The format of these specifications can be of any form as long as they are composed of only the *s*, *c*, and $+$ functions as given in Table 2. For adders that are not given in this table (e.g., 4:2 compressors), users may derive their specifications by simplifying them with the lemmas introduced below.

**Table 2.** Rewritten outputs of some adders

Adder	$out_3$	$out_2$	$out_1$	$out_0$
Half-adder	–	–	$c(a_0 + a_1)$	$s(a_0 + a_1)$
Full-adder	–	–	$c(a_0 + a_1 + a_2)$	$s(a_0 + a_1 + a_2)$
Vector adders	$$ \begin{array}{l} s(a_3 + b_3\\ \;\;+c(a_2 + b_2\\ \;\;\;\;+c(a_1 + b_1\\ \;\;\;\;\;\;+c(a_0 + b_0))) \end{array} $$	$$ \begin{array}{l} s(a_2 + b_2\\ \;\;+c(a_1 + b_1\\ \;\;\;\;+c(a_0 + b_0)))\\ \end{array} $$	$$ \begin{array}{l} s(a_1 + b_1\\ \;\;+c(a_0 + b_0 ) )\\ \end{array} $$	$$\begin{array}{l} s(a_0 + b_0 )\\ \end{array}$$

We expect adder modules to be composed of logical AND ($\wedge$), OR ($\vee$), XOR ($\oplus$), and NOT ($\lnot$) gates in certain patterns. We get expressions for these circuits’ functionality in terms of these functions through SVL semantics. We rewrite these expressions with the lemmas given below to simplify them to the same form as their specification. We define the operators $\wedge$ (and), $\vee$ (or), $\oplus$ (exclusive or), and $\lnot$ (negation) to work with integer-valued bits (e.g., $1 \wedge 0 = 0$, $1 \vee 1 = 1$, or $0 \oplus 1 = 1)$.

#### Lemma 1

$$ \forall x, y \in \{0,1\}\ x \oplus y = s(x + y)$$

#### Lemma 2

$$ \forall x, y \in \{0,1\}\ x\wedge y = c(x + y)$$

#### Lemma 3

$$ \forall x, y, h, g \in \{0,1\}\ c(x + y + h) \vee (s(x+y) \wedge g) = c(x + y + (h \vee g))$$

We implement these lemmas as well as some corollaries as rewrite rules so that terms that can be unified with the *lhs* of equations are replaced by their respective *rhs*. An example corollary is $\forall x, y, g \in \{0,1\}~(x~\wedge ~y)~\vee ~(s(x~+~y)~\wedge ~g) = c(x~+~y~+~g)$ that can be derived from Lemmas 2 and 3. Similarly, $\forall x, y, h \in \{0,1\}\ c(x + y + h) \vee s(x+y) = c(x + y + 1)$ can be derived from Lemma 3. These extra lemmas help expand our coverage to match more term patterns that may occur.

We add other rewrite rules using elementary properties of $\vee$, $\wedge$ and $+$ that help facilitate simplification. Lemma 3, and some corollaries rewrite terms with repeated variables. In such cases, in order for the rewriter to match the *lhs* with an applicable term, it is necessary to flatten the terms with associativity (e.g., $((a + b) + c) = (a + b + c)$) and lexicographically sort them using commutativity (e.g., $(b + a) = (a + b)$) for every $+$, $\vee$ and $\wedge$ instance. Other examples of rewrite rules we have in our system implement identity and inverse properties of addition. Finally, we have a lemma that rewrites the definition of $\oplus$, which is $(\lnot ab \vee a \lnot b)$, in terms of *s* as given in Lemma 1.

Note that we put a restriction on the use of the rewrite rule for Lemma 2 such that it is used only when *x* and *y* are input wires of the adder module. The function *c* is a specification for carry, and not all AND gates may calculate carry by themselves. We have observed that only the logical AND of input signals should be rewritten to *c*. Rewriting the other instances of $\wedge$ in terms of *c* prevents application of Lemma 3 and complicates our rewriting approach. We enforce this restriction in ACL2 through a syntactic check.

Our experiments given in Sect. 6 demonstrate that the method we described in this section can automatically simplify vector adders including ripple-carry, carry-lookahead [26] and parallel-prefix adders such as Brent-Kung [4], Ladner-Fischer [20], Kogge-Stone [19], Han-Carlson [9] and others.

Reasoning about adder modules before the candidate multiplier module is a crucial step in our rewriting mechanism. The functionality of all the adder modules should be represented with the *s*, *c*, and $+$ functions when expanding the definition of the multiplier module. Then, and only then, the multiplier design can be simplified and proved correct with the lemmas described in the subsequent section.

### Multiplier Module Proofs

After creating rewrite rules for adder modules, we start working with the correctness proof of our candidate multiplier design as given in Listing 1.1. Similarly, we convert multiplier modules into ACL2 expressions, replace instantiated adder modules with their specifications, and perform simplification with a rewriting mechanism derived from the lemmas introduced in this section. We first describe how we simplify complex expressions that originate from summation tree algorithms such as Wallace-tree. Secondly, we add more lemmas to simplify partial product logic that may be generated with Booth encoding. After rewriting with these lemmas, we expect to have simplified multiplier designs to our targeted final form as given in Table 1. We rewrite the multiplication specification into our final form as well and conclude verification with a syntactic equivalence check.

**Simplify Summation Trees.**

In some integer multiplier designs, summation of partial products may be implemented with a very irregular structure, as is the case with Wallace-tree like multipliers (see Sect. 2.3), and it can be challenging to simplify them to a regular and more easily interpretable form. We describe a set of lemmas, solving this problem by providing an efficient and automated mechanism for such complex structures. Below, we discuss the simplification method for multiplier designs implemented with simple partial products.

Having rewritten the adder components in terms of the *s*, *c*, and $+$ functions, Example 3 shows the term representing the 4th LSB of a Wallace-tree multiplier output. Our goal is to reduce such terms to our final form as given in Table 1.

#### Example 3

The 4th LSB of the Wallace-tree multiplier output from Fig. 1b after adder submodules are rewritten in terms of the *s*, *c* and $+$ functions:

$$\ \ \ \ \ s(\ s(\ s(a_3b_0 + a_2b_1 + a_1b_2)$$

$$\ \ \ \ \ \ \ \ \ \ \ \ +a_0b_3$$

$$\ \ \ \ \ \ \ \ \ \ \ \ +c(a_2b_0 + a_1b_1 + a_0b_2))$$

$$\ \ \ \ \ \ \ \ +c(s(a_2b_0 + a_1b_1 + a_0b_2) + c(a_1b_0 + a_0b_1)))$$

In such summation trees, we observe many nested calls for *s*. These can be simplified easily by the following rule.

#### Lemma 4

$$ \forall x, y \in \mathbb {Z}\ s(s(x) + y) = s(x + y)\ $$

#### Example 4

Example 3 simplified with Lemma 4:

$$\ \ \ \ \ s(a_3b_0 + a_2b_1 + a_1b_2 + a_0b_3$$

$$\ \ \ \ \ \ \ +c(a_2b_0 + a_1b_1 + a_0b_2)$$

$$\ \ \ \ \ \ \ +c(s(a_2b_0 + a_1b_1 + a_0b_2) + c(a_1b_0 + a_0b_1)))$$

Terms derived from summation trees may include many instances for addition of two or more calls of *c*. Since such instances are not present in the final form, we try to remove them. That can be done by merging such calls of *c* through a temporary conversion to *d* as implemented with the lemmas given below.

#### Definition 3

We define function *d* as follows.

$$\begin{aligned} \forall x \in \mathbb {Z} \ d(x) = {\frac{x}{2}} \end{aligned}$$

#### Lemma 5

$$ \forall x, y \in \mathbb {Z}\ c(x) + c(y) = d(x + y - s(x) - s(y)) $$

#### Lemma 6

$$ \forall x, y \in \mathbb {Z}\ c(x) + d(y) = d(x + y - s(x))\ $$

#### Lemma 7

$$ \forall x, y \in \mathbb {Z}\ d(x) + d(y) = d(x + y)\ $$

#### Lemma 8

$$ \forall x \in \mathbb {Z}\ d(-s(x) + x) = c(x)\ $$

Applying Lemmas 5, 6, 7, and 8 repeatedly to the term in Example 4, we obtain the term given in Example 5. Since $\forall a, b \in \{0,1\}\ c(a \wedge b) = 0$, we have a term that matches the 4th bit of the final form for multiplication as given in Table 1. It is not required to convert certain instances of *d* back to *c* with Lemma 8; however, we can achieve better proof-time performance by shrinking terms with this rewrite.

#### Example 5

Example 4 simplified with Lemma 5, 6, 7, 8:

$$ \ \ \ \ \ s(a_3b_0 + a_2b_1 + a_1b_2 + a_0b_3 $$

$$ \ \ \ \ \ \ \ \ +c(a_2b_0 + a_1b_1 + a_0b_2 $$

$$ \ \ \ \ \ \ \ \ \ \ \ \ \ + c( a_1b_0 + a_0b_1)))$$

Rewriting with Lemmas 5 and 6 creates new instances of *s*, which may not seem preferable at first glance because terms become less similar to the final form. However, we have found that for correct designs, these extra subterms cancel out and vanish during the rewriting process. We have seen this to be the case even for very large and much more complex terms that may have millions of nodes.

We implement these lemmas as rewrite rules as well as some elementary algebraic properties in order to flatten and sort terms lexicographically in summations. Our rewrite rules do not subsume each other, and they may be applied with an arbitrary order until none of the rules are applicable.

**Simplify Partial Products.** Unlike the simple partial product generation method, multipliers with Booth encoding implement a more advanced algorithm to generate partial products. That results in terms that are more complex (see Example 6) than those we have addressed so far. We expand our rewriting mechanism for simplification of summation trees and add more rewrite rules for automated simplification of partial products such as the ones generated with Booth encoding and sign extension tricks.

#### Example 6

Below is a term for the second LSB of a multiplier output, implemented with Booth radix-4 encoding and before any simplification for partial products took place:

$$\ \ \ \ s([\lnot b_1 b_0 a_1 \vee b_1 \lnot b_0 \lnot a_0 \vee b_1 b_0 \lnot a1]$$

$$\ \ \ \ \ \ \ \ +c([b_1 b_0 \vee b_1 \lnot b_0 ]$$

$$\ \ \ \ \ \ \ \ \ \ \ \ \ +[b_1 \lnot b_0 \vee \lnot b_1 b_0 a_0 \vee b_1 b_0 \lnot a_0 ]))$$

Similar to other multiplier verification methods [25], we perform algebraic rewriting on the $\oplus$, $\vee$ and $\lnot$ functions with the following lemmas.

#### Lemma 9

$$ \forall x \in \{0,1\}\ \lnot x = 1 - x $$

#### Lemma 10

$$\forall x, y \in \{0,1\}\ x \vee y = x + y - x y\ $$

#### Lemma 11

$$\forall x, y \in \{0,1\}\ x \oplus y = x + y - x y\ - x y\ $$

#### Example 7

Example 6 rewritten with Lemma 9, 10, and 11 as well as elementary algebraic properties.

$$\ \ \ \ s(b_1 + b_0 a_1 - b_1 a_0 + b_1 b_0 a_0 - b_1 b_0 a_1 - b_1 b_0 a_1$$

$$\ \ \ \ \ \ \ +c(b_1 + b_1 + b_0 a_0 - b_1 b_0 a_0 - b_1 b_0 a_0))$$

We would like such expressions to be simplified to our final form. When deriving our rewrite rules, we concentrate on the terms with negative and/or duplicate arguments and realize that applying the following set of lemmas is sufficient to simplify such complex expressions.

#### Lemma 12

$$\forall x, y \in \mathbb {Z}\ s((-x) + y) = s(x + y) $$

#### Lemma 13

$$\forall x, y \in \mathbb {Z}\ c((-x) + y) = (-x) + c(x + y) $$

#### Lemma 14

$$\forall x, y \in \mathbb {Z}\ d((-x) + y) = (-x) + d(x + y) $$

#### Lemma 15

$$ \forall x, y \in \mathbb {Z}\ s(x + x + y) = s(y) $$

#### Lemma 16

$$ \forall x, y \in \mathbb {Z}\ c(x + x + y) = x + c(y) $$

#### Lemma 17

$$ \forall x, y \in \mathbb {Z}\ d(x + x + y) = x + d(y)$$

#### Example 8

Below is the resulting term after Example 7 is simplified using Lemma 12–17 and elementary algebraic properties. We obtain a term matching the final form in Table 1.

$$\ \ \ \ \ s(b_0 a_1 + b_1 a_0 + c(b_0 a_0))$$

We implement these lemmas as rewrite rules along with the rules for simplification of summation trees. All of these lemmas automatically work together without any user intervention.

Algebraic rewriting of logical gates can be very expensive in terms of time and memory. For this reason, we limit the application of these rules to the partial product logic only. For example, if applied indiscriminately, Lemmas 10 and 11 can cause terms to grow exponentially. Even though partial product generation logic may allocate a large area in multipliers, rewriting the adders to the *s*, *c*, and $+$ functions isolates partial products from each other and segregates them into small chunks. We expect that expressions representing partial products are composed of the $\vee$, $\wedge$, $\oplus$, and $\lnot$ functions only. Therefore, we restrict Lemmas 9–11 to apply to terms that are composed of these functions only; and we restrict Lemmas 12–17 to apply to terms that are composed of minterms, and the − and $+$ functions only. For instance, in Lemma 13, if we are unifying *x* with a term that contains an instance of *s*, *c* or *d*, then we prevent rewriting with a syntactic check. This heuristic helps contain this potentially expensive approach to only local and smaller terms.

**Rewrite the Multiplier Specification.**

In our proposed rewriting scheme, we have a targeted representation for each output bit of multiplication as given in Definition 2. The rewriter cannot derive this form directly from the built-in ACL2 multiplication ($*$) function. Thus, we provide a recursively defined function *multbycol* that follows the formula in Definition 2. We prove *multbycol* to be equivalent to the $*$ function. When the rewriter works on the conjecture stating the correctness of a multiplier design as shown in Listing 1.1,  is rewritten to  . The rewriter can then efficiently convert the specification into the targeted final form.

Using the rewriting mechanism described in this section, we can verify multipliers with Baugh-Wooley, sign/unsigned Booth radix-4, and simple partial product generation algorithms with various summation tree algorithms such as Wallace and Dadda tree. Note that Lemmas 9–17 work together with Lemmas 4–8 but contradict Lemmas 1–3. This is the reason why our method relies on semantics where the design hierarchy is maintained so that we can simplify the logic in adder modules with Lemmas 1–3 and simplify the remainder of a multiplier design with Lemmas 4–17 at a different time. When this separation is possible, multiplier designs are verified fully automatically without requiring users to designate the type of algorithm used. The complete process of proving the equivalence of semantics of a multiplier design to its specification is verified using ACL2.

## Termination

Our rewriter does not enforce proof of termination for rewrite rules. The program terminates either when there are not any applicable rules or when a certain number of steps are taken, which may happen if that number is too small for the current conjecture, there is a loop between rules, or some rules grow some terms indefinitely. Even though it is not required by the rewriter, it is important to show that our rewriting algorithm requires a limited number of steps and does not run indefinitely.

Terms from conjectures change every time a rewrite rule is applied. Therefore, for each of our rewriting algorithms (adder and multiplier module simplification), we define a measure calculated on the term and show that it decreases every time we rewrite with one of our lemmas. We first define the measure for simplifying adder modules (Lemmas 1–3). Since carried out separately, we define another measure for the summation tree and partial product simplification algorithms (Lemmas 4–17). For brevity, we omit the discussion for termination with other lemmas pertaining to elementary algebraic properties such as commutativity and associativity.

### Measure for Adder Module Simplification

The first part of our multiplier verification algorithm is simplifying the logic in adder components and rewriting them in terms of the *s*, *c*, and $+$ functions. Below, we define auxiliary functions and a measure that guarantees termination of this part of the algorithm that rewrites terms with Lemmas 1–3.

#### Definition 4

**(**$f_1$ **).** Function $f_1$ counts the number of symbols (constants, functions and variables) in a term.

#### Definition 5

**(**$f_2$ **).** Function $f_2$ counts the occurrences of $\wedge$ and $\oplus$ in a term.

For example, computing $f_1$ and $f_2$ on the term $s( x \oplus y + x \wedge z + c( x \oplus y))$ yields 13 and 3, respectively.

#### Definition 6

We define a measure $m_1$ as follows, where the resulting ordered pairs are compared lexicographically.

$$m_1(term) = <f_2(term), f_1(term)>$$

The pairs produced by $m_1$ are ordered lexicographically: thus, the value of $m_1$ decreases if $f_2$ decreases (no matter the value of $f_1$), or $f_2$ stays the same and $f_1$ decreases. Rewriting with Lemmas 1, 2, and 3 decreases $f_2$. Rewriting with some corollaries does not change the value of $f_2$ but decreases $f_1$ . For example, rewriting with the corollary $\forall x, y, h \in \{0,1\}\ c(x + y + h) \vee s(x+y) = c(x + y + 1)$ does not change $f_2$ but decreases $f_1$. In short, every step taken with these lemmas decreases the value of $m_1$ calculated on the resulting term. Therefore, the rewriting algorithm for adder modules terminates.

### Measure for Multiplier Module Simplification

Rewriting for summation tree and partial product generation algorithms are performed together with a rewriting algorithm derived with Lemmas 4–17, excluding Lemmas 1–3. Therefore, we define a single measure to describe the termination of this part of the rewriting mechanism. Below we give definitions for some auxiliary functions and our measure.

#### Definition 7

**(**$f_3$ **).** Function $f_3$ sums the occurrence-depth of negative minterms, where the occurrence-depth is calculated with respect to the overall term.

For example, computing $f_3$ on the term $s(x_0x_1 + c( - x_2y_0 + c(- x_3y_1)))$ yields 5 because its negative minterms $- x_2y_0$ and $- x_3y_1$ occur at depth 2 and 3, respectively. These values can be calculated by counting the unclosed parentheses from the beginning up to the occurrence of these terms.

#### Definition 8

**(**$f_4$ **).** Function $f_4$ computes the number of unique occurrences of functions $\{c,\ d,\ \lnot ,\ \oplus ,\ \vee \}$.

For example, computing $f_4$ for the term $c(x_0) + s(x_1 + c(x_0) + c(x_1))$ yields 2 because even though there are three instances of *c*, the second occurrence of $c(x_0)$ is not counted.

#### Definition 9

We define measure $m_2$ to return ordered triples as follows, to be compared lexicographically.

$$m_2(term) = <f_4(term), f_3(term), f_1(term)>$$

The value of $m_2$ decreases if $f_4$ decreases, or $f_4$ stays the same and $f_3$ decreases, or $f_4$ and $f_3$ stay the same and $f_1$ decreases. Below we discuss how rewriting with Lemmas 4–17 satisfy this measure for termination.

Rewriting with Lemmas 4 and 8 does not change the value of $f_4$. For both lemmas, if *x* is unified with a term that contains a negative minterm, then the value of $f_3$ decreases, otherwise, $f_3$ remains the same. By removing an instance of *s*, rewriting with both lemmas decreases $f_1$ and consequently $m_2$.

Rewriting with Lemmas 5, 6, 7, 9, 10, and 11 decreases $f_4$, and therefore $m_2$, by removing an instance of *d*, *c*, $\lnot$, $\vee$ or $\oplus$. Even though rewriting with some of these lemmas creates copies of terms, the value of $f_4$ decreases because it does not count the same term more than once.

Rewriting with Lemmas 12–17 does not affect the value of $f_4$ since they are restricted to rewrite terms that contain only the $+$ and − functions, and minterms. For Lemmas 12, 13, and 14, *x* can only be unified with a positive minterm. Therefore, rewriting with these lemmas does not change $f_3$. For Lemmas 15, 16, and 17, if *x* is unified with a negative minterm, then $f_3$ decreases. Otherwise, $f_3$ remains the same and $f_1$ decreases.

In short, rewriting with Lemmas 1–3 decreases the measure $m_1$ and rewriting with Lemmas 4-17 decreases the measure $m_2$. Therefore, our proposed rewriting mechanism terminates.

## Experiments

In this section, we present our experimental results and compare them to the other state-of-the-art tools for the automated verification of multiplier designs. We have gathered a large set of multipliers from 3 different generators, and run all the experiments for other verification tools and ours on the same computer (A 2014 model iMac Intel(R) Core(TM) i7-4790K CPU @ 4.00 GHz with 32 GB system memory) for comparison. The instructions and a ready-to-run VM image to run our tool and reproduce these experimental results can be found online at http://mtemel.com/mult.html.

For benchmarking, we used 3 different generators. The tool from Homma et al. [10] generates Booth encoded sign and unsigned multipliers (input size up to 64 bits) with various summation tree and final stage adders. Designs from Homma et. al. have multiple copies of half/full-adder modules as well as some other adder modules. Since our method requires reasoning about each adder module, we wrote a function that scans the modules and automatically simplifies them as described in Sect. 4.1. Secondly, we used *SCA-genmul* [24] to generate simple unsigned and Baugh-Wooley based signed (also referred to as simple signed) multipliers. This tool does not generate Booth-encoded multipliers. Finally, we used another multiplier generator [34] that can generate large Booth-encoded multipliers.

**Table 3.** Proof-time results in seconds for various multiplier designs

Size	Benchmark	AM [23]$^\mathrm{a}$	DK [16]		Our tool	
		Unsigned	Unsigned	Signed	Unsigned	Signed
$64\times 64$	sca sp-dt-bk	39	6	6	1	1
	sca sp-wt-lf	33	6	6	1	1
	sca sp-cwt-ks	TO	65	58	1	1
	sca sp-ar-rc	23	5	5	1	1
	tem sp-dt-ks	173	7	7	1	1
	tem sp-wt-lf	33	6	6	1	1
	tem bp-dt-hc	TO	44	49	1	1
	tem bp-wt-rp	TO	45	49	2	2
	hom bp-dt-ks	288	8	TE	2	2
	hom bp-bdt-hc	TO	7	7	2	2
	hom bp-os-bk	71	6	TO	3	3
	hom bp-wt-cla	108	24	21	13	12
	hom bp-4:2-lf	TE	7	7	3	3
$128\times 128$	sca sp-dt-bk	643	33	36	2	3
	sca sp-wt-lf	633	34	38	2	2
	sca sp-cwt-ks	TO	TO	TO	3	3
	sca sp-ar-rc	384	27	27	18	18
	tem sp-dt-ks	TO	47	49	2	3
	tem sp-wt-lf	650	40	40	2	2
	tem bp-dt-hc	TO	877	1037	7	7
	tem bp-wt-rp	TO	918	1067	12	13
$256\times 256$	sca sp-dt-bk	TO	213	209	9	11
	sca sp-wt-lf	15351	226	223	11	13
	sca sp-cwt-ks	TO	TO	TO	13	15
	tem sp-dt-ks	TO	234	232	10	12
	tem sp-wt-lf	15552	220	221	10	12
	tem bp-dt-hc	TO	11555	14043	41	47
	tem bp-wt-rp	TO	11975	14264	54	58
$512\times 512$	sca sp-dt-bk	TO	1562	1562	53	64
	sca sp-wt-lf	TO	1588	1577	61	76
	tem sp-dt-ks	TO	1655	1655	68	75
	tem sp-wt-lf	TO	1604	1609	65	82
	tem bp-dt-hc	TO	TO	TO	246	281
	tem bp-wt-rp	TO	TO	TO	371	380
$1024\times 1024$	sca sp-dt-bk	TO	13746	13247	339	397
	sca sp-wt-lf	TO	13560	14005	322	345
	tem sp-dt-ks	TO	14125	15198	324	392
	tem sp-wt-lf	TO	13664	13708	327	393

$^\mathrm{a}$ Does not produce certificates.

**TE**: Terminated with an error. **TO**: Time-out. 5400 s. (90 min) for $64\times 64$ and $128\times 128$, 16200 s (270 min) for the rest.

We have measured the complete proof time for each benchmark, when available, and compared our results to the work of D. Kaufmann et al. [16] and A. Mahzoon et al. [23]. These methods are based on computer algebra, and they are the best performing tools at the time this paper is rewritten. Since we verified the correctness of our tool using ACL2, we do not generate certificates. D. Kaufmann et al. implement their method in a stand-alone C program but they generate certificates to check their proofs. We measured the total time to verify/certify and check certificates. A. Mahzoon et al. also test their method with a stand-alone C program but it does not produce any certificates. Even though it is not a complete comparison, we still include the results of their tool for the same benchmarks.

When we run our tool on these benchmarks, we only need to identify the names of the adder modules, their I/O size; multiplier I/O size, and whether they perform signed or unsigned multiplication in order to determine their specification. The proofs finish automatically, and users can see the specification explicitly to validate what is proved. The other tools are not interactive and use some heuristics to decide on the specification internally based on the design.

D. Kaufmann et al. [16] and A. Mahzoon et al. [23] both use AIGs as inputs, and we use SVL [33], all of which are translated from (System) Verilog using external tools. For the other tools, we used Yosys [39] and ABC [3] to create AIGs, without any optimization. For our tool, we created SVL netlists as described in Sect. 2.2. Since we compare the performance of different verification methods, we do not include the translation time in any of these results.

Table 3 shows the result of experiments run with a collection of circuits. The benchmarks are described with the generator, partial product generation algorithm, summation tree algorithm, and final stage adder. Generators are *tem* [34], *sca* [24], and *hom* [10]. Partial product generation algorithms are *sp* (simple unsigned/signed or Baugh-Wooley-based), and *bp* (unsigned and signed Booth radix-4 encoded). Summation tree algorithms are *dt* (Dadda tree), *wt* (Wallace tree), *cwt* (counter-based Wallace tree), *ar* (array), *os* (overturned-stairs tree), *bdt* (balanced delay tree), and *4:2* (4-to-2 compressor tree). Finally, the final stage adders are *bk* (Brent-Kung), *lf* (Ladner-Fischer), *rc* (Ripple-carry), *ks* (Kogge Stone), *csk* (Carry-skip), *hc* (Han-Carlson), and *cla* (Carry-lookahead). The selection of benchmarks was arbitrary but we have concentrated on Wallace-tree-like multipliers with complex final stage adders as they have a more widespread industrial application. For experiments with 64 $\times$ 64 and 128 $\times$ 128 multipliers, we set the time limit to 1.5 h, and for larger designs, we set the limit to 4.5 h. The results are given in seconds rounded to the nearest integer.

For all the benchmarks we have tested, our tool out-performed the other tools in all cases. Our method is shown to verify benchmarks the others cannot and produce a more homogeneous timing performance across different designs. A. Mahzoon et al. [23] work only on unsigned multipliers. Both A. Mahzoon et al. and D. Kaufmann et al. [16] give fluctuating results for multipliers with different architectures and/or different generators. For some benchmarks, the other tools terminated with an error such as segmentation fault (marked with TE). Our work is more resilient to differences in designs and it scales much better (proof times increase by 4.5–6 times when circuit size grows 4 times). For Wallace-tree like multipliers with simple partial products, about 40% of the time on average is spent on simplification with the lemmas given in Sect. 4, and the rest is spent by conversion of SVL semantics to ACL2 expressions. For multipliers with Booth-encoding, over 70% of the time is spent on partial product simplification. Array multipliers are the only type of circuit for which our tool struggles to scale. We believe that is because the minimal parallelism this circuit implements causes our rewriting engine to do much more work as compared to other multiplier structures. Even though memory use is not reported here, it scales the same way as timings, and it grows as big as 30 GB for the largest (1024 $\times$ 1024) circuits we have tested.

Additionally, since integer multipliers are used to implement floating-point operations, we tested our method in a correctness proof for an implementation of a floating-point multiply-add instruction for Centaur Technology, and we got similar results.

## Related Work and Conclusion

Having described our method, we now compare it with the related work. Well-known methods to verify multipliers include generic reasoning methods such as BDDs and SAT solvers. However, these tools do not scale well with large multipliers. For the last few years, efforts to verify large integer multipliers have explored the symbolic computer algebra approach based on Gröbner basis [7, 16, 22, 23, 28, 37]. As far as we are aware, all these tools are stand-alone, unverified C programs and none of them except D. Kaufmann et al. [16] produces certificates. The soundness and completeness of this approach is shown only in theory [17]. We compared our method to the studies with the best timing performance [16, 23]. The tools implementing these methods identify adder components in designs automatically and perform some rewriting. Their rewriting strategy is different than ours; their method does not rely on maintained design hierarchy and separate reasoning of adder and multiplier modules. Even though they provide a more automatic system, their application appears to be limited to some known patterns. Additionally, our tool is implemented on an interactive tool, which can enable users to carry out more complicated proofs such as the correctness of floating-point circuits. The limitation of our method is that it relies on maintaining circuit hierarchy. Should this pose a problem for some designs, it might be possible for our method to be adapted in the future to work with flattened modules and identify adder components similarly to the related work.

When a proof fails for a multiplier design, our tool does not output a user-friendly message. We will work to improve our tool to process the resulting terms from failed verification attempts and generate counterexamples for incorrect designs.

In this paper, we have presented an efficient method with a proven tool to verify large and complex integer multipliers. With maintained circuit hierarchy, we can automatically verify very irregular multiplier designs; for example, various $1024\times 1024$ Wallace-tree like multipliers can be verified in less than 10 min. We believe that our tool can find broader applications because it can be extended to verify circuits, such as floating-point multipliers, that include an integer multiplier as a submodule.

## Footnotes

1 By convention, “  ” characters surrounding variables, such as  , signify constants in ACL2.
